# *Fusarium* Mycotoxins and OTA in Beer from Shanghai, the Largest Megacity in China: Occurrence and Dietary Risk Assessment

**DOI:** 10.3390/foods12163071

**Published:** 2023-08-16

**Authors:** Anqi Xu, Haiyan Zhou, Shenghao Yu, Yiqi Li, Lan Wang, Aibo Wu, Jiang Liang, Shaojie Peng, Na Liu

**Affiliations:** 1 SIBS-UGENT-SJTU Joint Laboratory of Mycotoxin Research, CAS Key Laboratory of Nutrition, Metabolism and Food Safety, Shanghai Institute of Nutrition and Health, University of Chinese Academy of Sciences, Chinese Academy of Sciences, Shanghai 200031, China; xuanqi2021@sinh.ac.cn (A.X.); zhouhaiyan2018@sinh.ac.cn (H.Z.); wanglan@sibs.ac.cn (L.W.); abwu@sinh.ac.cn (A.W.); 2 Information Application Research Center of Shanghai Municipal Administration for Market Regulation, Shanghai 200030, China; yshaoyshao@163.com (S.Y.); 18917602951@163.com (Y.L.); cnpsj@sohu.com (S.P.); 3 NHC Key Laboratory of Food Safety Risk Assessment, Chinese Academy of Medical Science Research Unit (2019RU014), Department of Risk Assessment, China National Center for Food Safety Risk Assessment, No. 37, Guangqu Road, Chaoyang District, Beijing 100022, China

**Keywords:** mycotoxins, beer, contamination, UHPLC–MS/MS, risk assessment

## Abstract

Beer is susceptible to mycotoxin contamination originating from infected grains. It could be that mycotoxins are not completely removed during the brewing process and remain in the final product. Nevertheless, there have been no surveys of exposure to mycotoxin for Chinese inhabitants through beer consumption. This study aimed to investigate the presence of eight mycotoxins in 158 beer samples purchased in Shanghai, the largest megacity in China. The multiple mycotoxins determination was carried out using ultra-high performance liquid chromatography-tandem mass spectrometry (UHPLC-MS/MS). Our findings revealed that 48.1% (76/158) of the beer samples were contaminated with *Fusarium* toxins. Deoxynivalenol-3-glucoside (D3G) and zearalenone (ZEN) were detected in 34.81% and 16.46% of the total samples, respectively. The significant differences between D3G/ZEN contamination and various beer types were performed. Furthermore, this study performed a health risk assessment for Shanghai residents based on data for *Fusarium* toxins and ochratoxin A (OTA) present in beer for the first time. The results revealed that the 95th percentile dietary exposures of Shanghai residents did not pose any chronic or acute health risks, either individually or in combination. Dietary exposures to *Fusarium* toxins revealed different risk levels among residents. The cumulative health risk for women is higher than that for men at the same beer consumption. In addition, the acute risk of DONs exposure for adults deserves concern. The insights obtained from this study may be of assistance for beer manufacturers and governmental regulators to further develop beer monitoring and guarantee public health.

## 1. Introduction

The most popular alcoholic beverage consumed worldwide is beer. The amount of beer consumed worldwide in 2020 was 177.50 million kiloliters, or roughly 280.4 billion 633 mL bottles [1]. Since 2003, China has been the world largest beer consumer, and has remained so for 18 consecutive years. By 2020, beer consumption in China will reach 36.088 million liters (accounting for 20.3% of the world market share) [1]. Beer is one of the products most susceptible to mycotoxin contamination from contaminated raw materials (barley, wheat, hops, yeast, adjuncts). Mycotoxin contamination of grain is often caused by poisonous field fungal infections such as *Aspergillus*, *Fusarium*, *Penicillium*, and *Alternaria* spp. It is in these environments where fungi are exposed to environmental conditions sufficient to produce mycotoxins [2]. Therefore, beer can be contaminated with more than one mycotoxin at the same time. Mycotoxin contamination in beer can occur during all beer brewing processes, including barley reception, malting, milling, mashing, fermentation, maturation, and stabilization. Mycotoxins are highly heat stable and relatively soluble in water, and as such they are converted from grain to beer [3]. Moreover, some mycotoxins can be transferred to their metabolites during the brewing stage [4]. There have been few controlled and prospective studies that have performed the relationship of beer categorization and mycotoxin contamination. For the fermentation style, beer can be divided into lager beer (bottom fermentation) and ale beer (top fermentation). Commonly, the temperature for top fermentation is 18–25 °C; for bottom fermentation, the temperature is 7–15 °C for 7–9 days [5]. Here, we focused on four beer classifications: fermentation style, alcohol content, color, and origin.

Mycotoxin contamination of beer in different countries has been reported continuously. A study in Mexico detected 23 mycotoxins in 132 beer samples, and the occurrence rates of deoxynivalenol (DON), 3-acetyl-deoxynivalenol (3A-DON), deoxynivalenol-3-glucoside (D3G), and fumonisin B1 (FB1) were 14.8%, 3.3%, 9.8%, and 4.9%, respectively [6]. Four different mycotoxins were detected in 34 beer samples taken from the Tunisian market, including DON (56%, 1.6–6.7 ng/mL), 15-acetyl-deoxynivalenol (15A-DON, 6%, 12.7–15.6 ng/mL), OTA (3%, 2 ng/mL), and sterigmatocystin (STG, 6%, 4.9–11 ng/mL); 9% of positive samples presented the cooccurrence of DON and 15A-DON [7]. The mycotoxin-positive samples in Lleida had average concentrations of 15.06, 32.78, 31.28, 13.19, and 23.72 µg/L for ZEN, FB1, DON, D3G, and HT-2 toxin, respectively. Among them, ZEN had the highest content (65%) in beer, and the content range was 8.24–62.96 µg/L [8]. In Europe, DON and HT-2 toxins were found in 59.7% (24.5–47.7 µg/L) and 9.1% (24.2–38.2 µg/L) of 154 beer samples, respectively [3]. Moreover, apart from parent mycotoxins, modified mycotoxins have been identified from raw materials (malt, barely, wheat, maize) in beer [9]. Notably, D3G, the most commonly glycosylated metabolite of DON, is catalyzed by UDP-glycosyltransferase [10]. To date, the detection of mycotoxins has mostly focused on raw materials (wheat, barley, and maize) rather than high-value cereal products (beer) in China [11,12,13,14]. Unfortunately, the coexistence of *Fusarium* toxins and OTA in beer has received little attention in China, and there are few published data on the coexistence of masked mycotoxins in beer and associated coexposure risk assessments among Chinese consumers.

The presence of mycotoxins in food products poses a significant health risk to humans and can cause economic losses, even at low levels. A sensitive and reliable detection method is indispensable in order to guarantee the safety of the final product. However, the method for simultaneous mycotoxins determination in beer has been limited until now. Currently, ultra-high performance liquid chromatography-tandem mass spectrometry (UHPLC–MS/MS) is considered one of the most sensitive and selective techniques for the simultaneous analysis of mycotoxins [5]. UHPLC-MS/MS has faster chromatographic separation, increased sensitivity, and improved resolution. For the health-based guidance values of risk assessment, the tolerable daily intake (TDI) and acute reference dose (ARfD) values were used as health guidance values for the chronic and acute exposure assessment. The Joint FAO/WHO Expert Committee on Food Additives (JECFA) set the TDI values for DONs (DON, 3A-DON,15A-DON, D3G), nivalenol (NIV), FB1, zearalenone (ZEN), and OTA to 1.0, 0.7, 2, 0.25, and 0.01 µg/kg·bw/d, respectively [15]. The European Food Safety Authority (EFSA) set the ARfD for DONs at 8 µg/kg·bw [16,17,18]. However, individual assessments of mycotoxins may underestimate their toxicity when they occur simultaneously. Several researchers have showed the health risk of mycotoxins in beer for inhabitants in Mexico [6], Tunisia [7], Spain [8], Poland [19], Latvia [20], and some other European countries [3]. However, there is a scarcity of data on exposures to mycotoxins through beer consumption for populations in China.

The purposes of the study are (i) to provide contamination data on mycotoxins in beer from the Shanghai market based on an improved and validated UHPLC–MS/MS method and (ii) to assess and rank the health risks by point and probability evaluations and perform the correlation between mycotoxin exposure and Shanghai residents’ age, gender, dietary habits, and other factors. This study fills the research gap on the coexistence of masked mycotoxins in beer and associated coexposure risk assessment among Chinese consumers. We believe that our study provides a reference and warning in the scope of beer safety for customers, beer manufacturers, and governments.

## 2. Materials and Methods

### 2.1. Chemicals and Reagents

DON (D0156), D3G (32911), 3A-DON (A6166), 15A-DON (A1556), NIV (34131), ZEN (Z2125), FB1 (F1147), and OTA (O1877) were purchased from Sigma products (St. Louis, MO, USA). The purity of the standard products is above 99%. UHPLC grade of formic acid (FA), acetonitrile (ACN), acetic acid (AA), methanol, and ammonium acetate were purchased from Merck (Darmstadt, Germany). Milli-Q quality water (18.2 MΩ·cm) was utilized throughout the study (Millipore, Billerica, MA, USA).

### 2.2. Sampling and Sample Preparation

In 2022, 158 various beer samples were acquired from supermarkets and beer shops in 7 different districts of Shanghai. To promote the discussion and comparison of the results, the samples were divided into the following categories: fermentation style—ale (n = 73) and lager (n = 85); alcohol content—nonalcoholic (n = 4), between 3 and 5% vol. (n = 87), and >5% vol. (n = 67); color—pale (n = 23), amber (n = 117), and dark (n = 18); and origin—domestic (n = 121) and foreign (n = 37). Prior to analysis, all beer samples were stored at 4 °C in a dry and ventilated environment. The descriptions of the contaminated samples are organized in Table S1.

Sample pretreatment for each beer was based on a previous study with minor modifications [8]. An appropriate amount of beer was ultrasonically degassed for 30 min (Geneng G-100S, Shenzhen, China). Then, 10 mL of extraction agent, consisting of ACN:water:AA (70:29:1, *v*/*v*/*v*), was added to 5 ± 0.1 mL of the degassed beer sample. The mixture was vigorously shaken for 30 s and fully ultrasonically extracted for 30 min (Kylin Bell QL 861, Haimen, China). After centrifugation at 5000 rpm for 10 min (Thermo Fisher SL 16R, Waltham, MA, USA), the supernate was filtered through a 0.22 μm organic filter membrane prior to injecting into the UHPLC system. The determination was made using external standard approach.

### 2.3. UHPLC–MS/MS Analysis and Validation

An ExionLC™ AD system coupled to the SCIEX Triple Quad™ 3500 mass spec system (AB SCIEX, Milford, MA, USA) was utilized. Analytes were separated on a CNW Athena C18 Column (1.8 μm, 2.1 × 100 mm). The mobile phases were made up of 0.1% acetic acid in water (A) and 100% methanol (B) gradient elution with a flow rate of 0.25 mL/min. The elution schedule was as follows: 0–2 min 5% B, 2–12 min 5–95% B, 12–12.1 min 95–99% B, 12.1–14 min 99% B, 14.0–14.1 min 99–5% B, and 14.1–16 min 5% B. The optimized parameters for multiple reaction monitoring (MRM) mode and positive (ESI+ 5.5 kV) and negative (ESI- 4.5 kV) electrospray ionization modes using mass spectrometry are source temperature 550 °C; air curtain gas pressure 35 psi; atomizing gas pressure 55 psi; aux gas pressure 60 psi; collision gas pressure 9 psi. The column was maintained at 35 °C. The capillary voltage was 550 V, and the spray gas was nitrogen. Nitrogen flow rates for atomizing and drying were 50 L/min and 10 L/min, respectively. The LC–MS/MS acquisition parameters are listed in Table 1.

Methods for correcting matrix effects include isotope internal standard and matrix standard curve methods. We used the latter method for quantification. Blank beer sample matrix was selected to be spiked with a series of 8 mycotoxin mixed standard solutions and pre-treated together with the samples. A linear equation was fitted with concentration as *x*-axis and peak area as *y*-axis and used for quantification of the results. For recoveries, the spiked concentrations were as follows: NIV, 300, 600 and 1500 µg/L; OTA, 3, 6, and 15 µg/L; others mycotoxins (DON, D3G, 3A-DON, 15A-DON, ZEN, and FB1), 30, 60, and 150 µg/L. The method was verified according to the guidelines of the document [21]. The details were listed in Table 2.

### 2.4. Beer Consumption Data

The population groups considered in this study were consistent with a previous study [13]: total population, adult men, adult women (Table S2). Currently, there is a lack of research on the correlations between beer consumption and various demographic factors, such as age, gender, and regional location, among the population in China. Therefore, the average alcoholic beer consumption per capita of 28.55 L in China in 2021, equivalent to 0.078 L/per-capita/day, was used.

### 2.5. Assessment of Health Risk

The current study adopts deterministic estimation (point estimation) and probabilistic estimation (Monte Carlo assessment model) to analyze health dietary risk. Three situations were applied for dietary exposure evaluation in consideration of data treatment of samples with no detection (<LOD). The values of 0, ½ LOD, and LOD were utilized as substitutions for lower, middle, and upper bounds, respectively [22]. In this study, we mainly focused on the upper bound data.

#### 2.5.1. Deterministic Estimation

This study employs beer mycotoxin contamination levels and consumption data to estimate the dietary exposure risk. Equation (1) reveals the calculation of estimated daily intake (EDI, µg/kg bw/day) [23]:

EDI = (C × C_A_)/B_W_ (1)

where:

C = average concentration of mycotoxins observed in beer (µg/L)

C_A_ = beer consumption (g·person^−1^·day^−1^)

B_W_ = body weight (kg)

#### 2.5.2. Probabilistic Estimation

Monte Carlo estimation was performed, utilizing @-Risk Industrial 7.5 application. Probability assessment was selected for its exposure factor variability and certainty compared with deterministic estimation [24]. The intrinsic variability combined with mycotoxin contents in beer as well as food consumption patterns were considered. After 10,000 iterations via Monte Carlo simulation, a steady exposure distribution with a confidence interval greater than 90% was obtained. To describe the statistics of the exposure simulation profile, the following parameters were used: mean, P50, P90, P95.

#### 2.5.3. Risk Characterization

For risk characterization, the hazard quotient (HQ) is applied by the ratio of the EDI and TDI (Equation (2)). The hazard index (HI) approach is employed in most mixture risk assessment (MRA). For calculation convenience, the sum of the HQs for the *Fusarium* toxins was obtained as HI (Equation (3)). Conventionally, an HI/HQ less than 1.0 was considered admissible, and citizens are less likely to access to a toxic level with probably health consequences. Otherwise, adverse effects would occur.

HQ = EDI/TDI(2)

HI = HQ DONs + HQ NIV + HQ ZEN + HQ FB1(3)

For OTA, considering its underlying neoplastic and nonneoplastic toxicity, the margin of exposure (MOE) approach, combined with the benchmark dose (BMD), was employed [25]. BMDL10 values of 14.5 (neoplastic). and 4.73 (nonneoplastic) µg/kg·bw/day were observed. MOE_1_ and MOE_2_ values greater than 200 and 10,000, respectively, suggested less health concern [17].

MOE_1_ = nonneoplastic_BMDL10/EDI (4)

MOE_2_ = neoplastic_BMDL10/EDI (5)

### 2.6. Data Analysis

Data was performed as mean ± standard error of the mean (SEM). Non-parametric tests were evaluated to compare the distribution features and differences for all beer samples at a significance level of 0.05, such as Mann–Whitney and Kruskal–Wallis test. All data management and drawings were implemented via GraphPad Prism 8.0 software (GraphPad software, San Diego, CA, USA).

## 3. Results and Discussion

### 3.1. Method Validation

Few previous studies in China have investigated the co-occurrence of *Fusarium* toxins and OTA in beer, and there has been little determination of appropriate methods. Table 2 details the validation parameters of the UHPLC-MS/MS method. The correlation coefficient (R^2^) of *Fusarium* toxins and OTA ranged from 0.9873 to 0.9998. the LOD and LOQ for mycotoxins were found to be in the range of 0.5–50 µg/L and 1.5–150 µg/L, respectively. They are lower than the given maximum levels for individual mycotoxins in foodstuffs [26]. The recoveries for every target analyte were measured at three spiked concentrations (low, middle, and high) based on vacant beer matrices. The recoveries were in the extent of 80.2–121.9%, and the RSDs were less than 20%. Taking these validation results together, the utilized method was reliable and precise for the detection of *Fusarium* toxins and OTA in various beer samples.

### 3.2. Mycotoxin Occurrence in Beer Samples

The contamination levels of the eight mycotoxins (DON, D3G, 3A-DON, 15A-DON, NIV, FB1, ZEN, and OTA) in beer samples in Shanghai are shown in Table 3. From the pool of 158 analyzed samples, only D3G and ZEN were detected, and the other six mycotoxins (DON, 3A-DON, 15A-DON, NIV, FB1 and OTA) were below the LOD in total beer samples. The cooccurrence of D3G and ZEN was detected in five beer samples. D3G and ZEN are mainly produced by *Fusarium* fungi, which are distinguished by infecting grains in the field [6].

The most prevalent contaminant was D3G (positive rate of 34.81%), with concentrations ranging from 5 to 495.24 µg/L. The average concentration levels (30.6 µg/L) reported here were relatively high when compared to previous data from Poland (3.5 µg/L for D3G) [27], and Europe (3.8 µg/L for D3G) [28]. Mycotoxin contamination is highly influenced by numerus internal and external factors, including geographical and environmental situations during distribution, storage, and sales; this might explain the contamination differences in different regions. Varga et al. [29] reported that concentrations of D3G and DON in beer were 6.9 µg/L and 8.4 µg/L in Austria and Germany, respectively. Wall-Martinez, Pascari, Ramos, Marin, and Sanchis [6] found that DON, D3G, and 3A-DON were observed in 87.5% of the contaminated beer samples purchased in Veracruz.

The reason why we only detected D3G rather than DON in beer samples was probably due to high D3G contaminated raw materials and DON conversion in malting conditions. On the one hand, the high-efficiency expression of UDP-glycosyltransferase (UGT) in grain could detoxify DON to D3G prior to harvest, thus allowing grain resistant to DON-producing pathogens [30]. On the other hand, processing conditions promote the transform of free mycotoxins (DON) into modified mycotoxins (D3G) [5]. It was reported that there was a significant increase in D3G content and a large reduction in DON, 3A-DON, and 15A-DON content during the malting process [7,30]. Malting consists of three steps: steeping, germination, and kilning. The DON content was reduced during steeping of barley, suggesting a wash effect due to water solubility [31]. As for germination, an increase in D3G was observed because of the enhancement in water activity in the cereals [32]. More importantly, glycosylation of parent mycotoxins (DON) may occur, and enzymatic activity can liberate mycotoxins connected to polysaccharides, thus generating a new compound (D3G). In regard to kilning, D3G increased while DON remained since it is thermostable [32]. In summary, these findings demonstrated our observations, which indicated that beer products had greater levels of D3G contamination while DON contamination was either low or undetectable.

ZEN contamination was found in 26 analyzed beer samples (positive rate of 16.46%), with concentrations ranging from 5.38 to 98.76 µg/L. The average concentration levels were 6.83 µg/L. It should be noted that highest positive sample (#57) was 98.76 µg/L. This is probably because the raw materials included a higher percentage of maize than other samples (Table S1). Maize has been proven susceptible to infection by ZEN-producing *F. graminearum* and *F. culmorum* [5]. Combined with the contamination results, it could be speculated that malting situations can accelerate the production of ZEN. In addition, the absorption, biodegradation, or conversion of ZEN by yeast during fermentation is likely to occur. Wall-Martínez et al. [33] reported a decrease in the content of ZEN in the wort by *Saccharomyces pastorianus* (lager beer yeast) (39–67%), which attributed to high adsorption to yeast. However, the risk of ingesting mycotoxins still remain because the yeasts in some beers are not removed. Zhang et al. [34] found that ZEN was degraded by *Saccharomyces cerevisiae*, probably due to the production of enzymes or proteins. In addition to absorption and biodegradation phenomenon, yeast was able to convert ZEN into its derivatives [35].

Considering fermentation style, the contamination level of D3G in ale beer (32.15 µg/L) was significantly higher than that in lager beer (29.28 µg/L) (Table S3). The reason can probably be attributed to variations in temperature and the adsorption of mycotoxins to the yeast cell throughout fermentation. Our results were consistent with a previous study [33] that reported that lower fermentation temperatures weakened the absorption of DON to yeasts. In addition, we did not detect ZEN in ale beer; this was attributed to the highest decrease in ZEN in the wort during the ale fermentation process [33]. On the other hand, the higher the alcohol level was, the more susceptible the beer was to mycotoxin contamination. A possible explanation for this might be that in order to achieve high alcohol levels, more cereals was utilized to produce denser wort, resulting in higher mycotoxin accumulation. This finding was also reported in [8]. Regarding beer color, the D3G content in dark beer (47.17 µg/L) was higher than in pale (26.19 µg/L) and amber (28.92 µg/L) beer (Table S3). This might be related to differences in the malting process that affect toxin distribution. Dark beer samples are mainly made with roasted malts, such as dark and burnt malts, which have a higher roasted temperature and a stronger burnt malt flavor. For beer origin, there are no significant differences between domestic and foreign beer. The D3G content in China (domestic) and foreign beer were 39.59 and 27.85 µg/L, respectively, and ZEN content in China and foreign beer were 5.87 and 7.12 µg/L, respectively (Table S3). Taken together, the differences between D3G/ZEN contamination and various types of beer (fermentation style, alcohol content, color) were demonstrated. More importantly, for governmental regulators and beer manufacturers, the health of the population is more likely to be ensured by proper fermentation temperature, reduced alcohol content, and lighter colored beer.

### 3.3. Risk Assessment and Characterization

#### 3.3.1. Chronic Risk Assessment of Individual Mycotoxin

EDI of mycotoxins in beer samples for populations in Shanghai, performed by deterministic and probabilistic estimation, are shown in Table 4. When it comes to chronic exposure assessment, the EDI values in the mean, 50th, 90th, and 95th percentiles were less than their TDI values in deterministic and probabilistic estimation (Table 4). In other words, the HQ values were all within 1, revealing no chronic health concern for the populations in Shanghai. The deterministic estimation showed that all EDI values ranking of mycotoxins in Shanghai residents decreased in the order of: DONs > NIV > ZEN > OTA = FB1. The EDI value ranking of the populations was as follows: adult women > total population > adult men. Interestingly, the EDI values ranking of mycotoxins by probabilistic estimation, as well as the ranking of populations, were consistent with the ranking results of deterministic estimation.

It is clear that DONs showed a predominated daily intake of mycotoxins. Despite not being the most hazardous, the DON group is the major source of substances harmful to human health. In addition, a visible downward trend could be seen with increasing age. It could be speculated that teenagers are more sensitive to mycotoxin exposure than adults. This finding was also reported by previous research, which found that children had higher daily exposures of DON than general population [13,36].

Due to the nonneoplastic and neoplastic effects of OTA, the MOE method was performed (Table 5). MOE_1_ and MOE_2_ surpassed 200 and 10,000 in different exposure scenarios, respectively, revealing less health risk. Combined with the results of individual mycotoxin risk assessments, the health of heavy drinkers and children should be examined, and urgent measures should be implemented to monitor and manage mycotoxins in beer.

#### 3.3.2. Cumulative Chronic Risk Assessment of *Fusarium* Toxins

For the cumulative risk assessment of numerous mycotoxins, the concentration addition (CA) concept was utilized, which assumes that mycotoxins act in the uniform way, only varying in the concentrations for provoking their toxic effects [37]. *Fusarium* toxins were grouped for the cumulative assessment.

**Figure 1:**
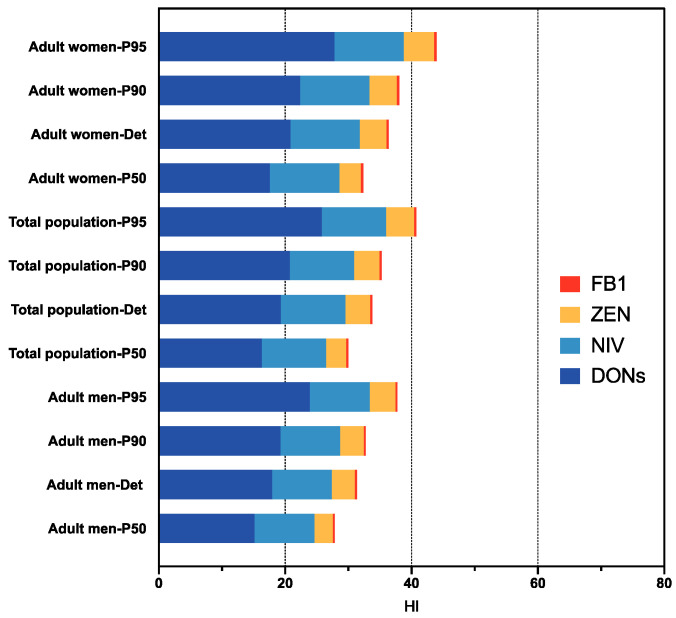
Cumulative risk assessment of *Fusarium* toxins (DONs, NIV, ZEN, FB1) through beer consumption were described in the upper bound deterministic (Det) and probabilistic estimation (P50, P90 and P95) for populations in Shanghai.

As mentioned above, individuals are exposed to numerous mycotoxins simultaneously, although risk assessment is typically conducted on single substances, which may bring about risk undervaluation. Hence, the cumulative health risks of a combination of *Fusarium* mycotoxins, including DONs, NIV, ZEN, and FB1, were assessed (Figure 1). The HI values in P95 were 0.44 (adult women), 0.41 (total population), and 0.38 (adult men). Consequently, combined exposure to *Fusarium* toxins through beer consumption is unlikely to cause health problems.

Considering the influence of sex differences, for all age groups, the underlying health risks for females were greater than those for males at the same level of consumption. There are no significant differences between females and males. This might be due to diverse dietary habits and lower body weight, resulting in higher risks. Huang et al. [38] found that the prevalence of DONs and ZEN of underweight members was exceeded the normal and overweight members. Regarding age differences, children are faced with higher mycotoxin exposure levels owing to progressive development of their body system and inferior detoxification capability when compared to adults [36]. Therefore, further work is required by the government and the public to guarantee children’s health in the face of cumulative mycotoxin exposure.

#### 3.3.3. Acute Risk Assessment of DONs

ARfD value is the amount of food and other substances ingested by humans during a period of 24 h or less that does not induce a detectable health risk to the individuals [16]. For acute exposure to DONs, P95 and P99 are generally considered as the threshold of concern in acute assessment [39]. In our study, the P95 exposure of DONs was less than the ARfD value (8 µg/kg bw). Thus, there is no toxicological concern associated with acute DONs exposure for 95% of the population from the probabilistic estimation (Table 4).

To elucidate a worst acute situation, the amount of beer a person would have to consume to reach 8 µg/kg·bw (ARfD) was assessed based on the highest concentrations (#13, 495.2 µg/L). An adult man weighing 62.7 kg would need to drink 1013 mL beer (approximately 2 bottles), to beyond the 8 µg/kg·bw (ARfD). For an adult woman weighing 54 kg, consuming 872 mL (approximately 1.7 bottles) would have the acute toxicity risk of DONs. Therefore, it is worth mentioning that the acute health risk of people with high alcohol consumption (heavy drinkers) are of concern.

## 4. Conclusions

This study has developed and validated a determination method of multiple mycotoxins in beer based on UHPLC-MS/MS. Of the 158 tested beer samples, D3G and ZEN were found in 34.81% and 16.46% of the total beer samples, respectively. Five samples were contaminated with D3G and ZEN simultaneously. D3G was detected rather than DON in beer samples purchased in Shanghai probably due to raw materials geographical and environmental situations in 2022. Malting situations probably accelerate the production of D3G and ZEN. Moreover, the differences between D3G/ZEN contamination and various types of beer (fermentation style, alcohol content, color) were demonstrated. And we have outlined that appropriate fermentation temperature, lower alcohol and lighter colored beer are more likely to ensure residents’ health. For beer origin, there are no significant differences between domestic (China) and foreign beer. This project is the first comprehensive health risk assessment of *Fusarium* toxins and OTA present in beer in China. No chronic dietary intake risk to the populations in Shanghai was observed at the 95th percentile. Nevertheless, for an adult man (62.7 kg), consumption of approximately 2 bottles of beer per day (500 mL per bottle) would exceed the ARfD value, which indicated that acute toxicity risk of DONs might occur. Furthermore, beer manufacturers should increase the gatekeeping of raw material procurement, and the governmental regulators improve the monitoring of mycotoxins and fill the gap in mycotoxin limit standards in beer in China.

## Figures and Tables

**Table 1:** LC-MS/MS parameters for the analysis of mycotoxins.

Mycotoxins ^1^	Molecular Weight	RT ^2^ (min)	Molecular Ion	ESI	Parent Ion (*m*/*z*)	Product Ions (*m*/*z*)	CE (eV)
DON	296.32	5.08	[M + H]^+^	+	297.10	249.20/203.00	16/22
D3G	458.50	5.30	[M − H]^−^	−	457.10	427.20/247.30	20/28
3A-DON	338.35	7.13	[M − H]^+^	+	339.10	231.00/203.10	16/19
15A-DON	338.35	7.14	[M − H]^−^	−	339.10	137.20/321.10	12/16
NIV	312.30	3.78	[M − H]^−^	−	310.90	281.00/205.00	14/24
ZEN	318.36	10.60	[M − H]^−^	−	317.20	175.00/273.00	33/27
FB1	721.00	9.00	[M − H]^+^	+	722.40	352.40/334.30	53/54
OTA	403.81	10.80	[M − H]^−^	−	402.10	358.00/166.80	29/46

^1^ DON = deoxynivalenol, D3G = deoxynivalenol-3-glucoside, 3A-DON = 3-acetyl-deoxynivalenol, 15A-DON = 15-acetyl-deoxynivalenol, NIV = nivalenol, ZEN = zearalenone, FB1 = fumonisins B1, OTA = ochratoxin A; ^2^ RT = Retention time.

**Table 2:** Overview of the methodological characteristics including Linearity range, correlation coefficient (R^2^), limits of detection (LOD); limits of quantification (LOQ) and recovery for the analysis of mycotoxins in beers.

Mycotoxins	Linearity Range (µg/L)	Correlation Coefficient (R^2^)	LOD (µg/L)	LOQ (µg/L)	Recovery ± RSD (%, n = 6)		
					Spiked Low	Spiked Middle	Spiked High
DON	15–250	0.9996	5	15	121.9 (10.0)	91.1 (3.1)	94.4 (4.2)
D3G	15–100	0.9965	5	15	109.1 (13.5)	104.8 (12.2)	82.1 (7.1)
3A-DON	30–500	0.9911	10	30	119.2 (11.1)	103.3 (2.3)	106.0 (1.0)
15A-DON	30–500	0.9873	10	30	115.8 (10.6)	102.2 (2.8)	89.8 (1.8)
NIV	150–2500	0.9990	50	150	80.7 (10.6)	104.0 (3.4)	88.9 (3.8)
ZEN	15–250	0.9941	5	15	111.6 (5.7)	112.6 (2.8)	80.6 (3.2)
FB1	15–250	0.9947	5	15	110.4 (15.7)	86.7 (9.7)	80.2 (5.4)
OTA	1.5–25	0.9998	0.5	1.5	121.6 (3.2)	92.0 (2.4)	88.1 (1.4)

**Table 3:** Contamination levels (µg/L) of eight mycotoxins in beer samples from Shanghai, China, in 2021 (n = 158).

Mycotoxin	MRLs ^1^	Below MRLs	Incidence	Positive Number	Mean	SD	Range
DON	1000 ^2^, 750 ^3^	100%	<LOD	<LOD	<LOD	<LOD	<LOD
D3G		100%	34.81%	55/158	30.60	54.59	5–495.24
3A-DON		100%	<LOD	<LOD	<LOD	<LOD	<LOD
15A-DON		100%	<LOD	<LOD	<LOD	<LOD	<LOD
NIV	NF	100%	<LOD	<LOD	<LOD	<LOD	<LOD
ZEN	60 ^2^, 75 ^3^	99.36%, 99.36%	16.46%	26/158	6.83	8.72	5.38–98.76
FB1	NF, 800 ^3^	100%	<LOD	<LOD	<LOD	<LOD	<LOD
OTA	5 ^2^, 3 ^3^	100%	<LOD	<LOD	<LOD	<LOD	<LOD

^1^ MRLs, Maximum Regulation Limits; SD, Standard Deviation; NF, Not Found. ^2^ MRLs for DON, ZEN, FB1, OTA in cereals and their products (GB 2761-2017). ^3^ MRLs for DON, ZEN, FB1, OTA in cereal flour, maize-based breakfast cereals, or processed cereals as end product marketed for direct human consumption (EC Regulation 2023/915).

**Table 4:** Estimated dietary intake (EDI) of mycotoxins in beer samples for populations in Shanghai, performed by deterministic estimation and probabilistic estimation at the upper bound (µg/kg·bw/day).

Mycotoxins	PMTDI (µg/kg bw/Day)	ARfd (µg/kg bw/Day)	Population	Deterministic Estimation	Probabilistic Estimation			
				Mean	Mean	P50	P90	P95
DONs	1	8	Total population	1.93 × 10^−1^	2.00 × 10^−1^	1.63 × 10^−1^	2.07 × 10^−1^	2.58 × 10^−1^
			Adult men	1.80 × 10^−1^	1.86 × 10^−1^	1.51 × 10^−1^	1.93 × 10^−1^	2.39 × 10^−1^
			Adult women	2.08 × 10^−1^	2.16 × 10^−1^	1.76 × 10^−1^	2.24 × 10^−1^	2.78 × 10^−1^
NIV	0.7	N.F.	Total population	7.13 × 10^−2^	7.13 × 10^−2^	7.13 × 10^−2^	7.13 × 10^−2^	7.13 × 10^−2^
			Adult men	6.62 × 10^−2^	6.62 × 10^−2^	6.62 × 10^−2^	6.62 × 10^−2^	6.62 × 10^−2^
			Adult women	7.69 × 10^−2^	7.69 × 10^−2^	7.69 × 10^−2^	7.69 × 10^−2^	7.69 × 10^−2^
ZEN	0.25	N.F.	Total population	9.74 × 10^−3^	8.35 × 10^−3^	7.89 × 10^−3^	9.99 × 10^−3^	1.11 × 10^−2^
			Adult men	9.04 × 10^−3^	7.76 × 10^−3^	7.33 × 10^−3^	9.27 × 10^−3^	1.03 × 10^−2^
			Adult women	1.05 × 10^−2^	9.00× 10^−3^	8.51 × 10^−3^	1.08 × 10^−2^	1.19 × 10^−2^
FB1	2	N.F.	Total population	7.13 × 10^−3^	7.13 × 10^−3^	7.13 × 10^−3^	7.13 × 10^−3^	7.13 × 10^−3^
			Adult men	6.62 × 10^−3^	6.62 × 10^−3^	6.62 × 10^−3^	6.62 × 10^−3^	6.62 × 10^−3^
			Total population	7.69 × 10^−3^	7.69 × 10^−3^	7.69 × 10^−3^	7.69 × 10^−3^	7.69 × 10^−3^
OTA	0.01	N.F.	Total population	7.13 × 10^−3^	7.13 × 10^−3^	7.13 × 10^−3^	7.13 × 10^−3^	7.13 × 10^−3^
			Adult men	6.62 × 10^−3^	6.62 × 10^−3^	6.62 × 10^−3^	6.62 × 10^−3^	6.62 × 10^−3^
			Adult women	7.69 × 10^−3^	7.69 × 10^−3^	7.69 × 10^−3^	7.69 × 10^−3^	7.69 × 10^−3^

N.F.: not found. Contamination levels below the LOD were considered as LOD (upper bound) for mycotoxin dietary exposure assessment.

**Table 5:** The estimation of OTA exposure in beer samples for populations in Shanghai, performed by deterministic and probabilistic estimation at the upper bound.

Method	Population	MOE_1_	MOE_2_
Deterministic estimation	Total population	6.63 × 10^5^	2.03 × 10^6^
	Adult men	7.15 × 10^5^	2.19 × 10^6^
	Adult women	6.15 × 10^5^	1.89 × 10^6^
Probabilistic estimation	Total population	6.63 × 10^5^	2.03 × 10^6^
	Adult men	7.15 × 10^5^	2.19 × 10^6^
	Adult women	6.15 × 10^5^	1.89 × 10^6^

## Data Availability

Data is contained within the article.

## Supplementary Materials

The following supporting information can be downloaded at: https://www.mdpi.com/article/10.3390/foods12163071/s1.
