# Supplementary material for: Fusarium Mycotoxins and OTA in Beer from Shanghai, the Largest Megacity in China: Occurrence and Dietary Risk Assessment
Source: Foods. 2023 Aug 16;12(16):3071. doi: 10.3390/foods12163071 (PMC10452965; doi:10.3390/foods12163071)
Supplement: Supplementary file 1 [file foods-12-03071-s001.zip › foods-2488113-supplementary.pdf]

Supplementary data

Fusarium mycotoxins and OTA in beer from Shanghai, the largest megacity in China: Occurrence and dietary risk assessment

Table S1 Description of contaminated beers purchased in Shanghai, China (n=76).

Sample	D3G (μg/kg)	ZEN (μg/kg)	Country of origin	Alc. Content (% vol.)	Original wort concentration (°P)	Fermentation type	Malt type	Unmalted adjuncts
1	N.D.	52.842	China	≥4.8	12	Lager	Barely	Maize
2	106.892	5.382	China	≥3.3	9	Lager	Barely, wheat	Pectin
4	83.163	N.D.	China	≥4.6	12	Lager	Barely, wheat	osmanthus tea
6	126.429	N.D.	China	≥5.5	13	Lager	Barely, wheat	-
7	83.951	N.D.	China	≥3.3	9	Lager	Barely	-
9	73.544	N.D.	China	5	11.2	Lager	Barely	Rice, maize
10	66.895	N.D.	China	5	11.2	Lager	Barely	Rice, maize
11	48.469	9.399	Denmark	5.2	12.2	Lager	Barely, wheat	Flavedo, coriander
12	350.753	N.D.	Ukraine	4.9	11.3	Lager	Wheat	-
13	495.243	N.D.	Ukraine	4.8	11.5	Lager	Barely, wheat	-
17	56.019	N.D.	China	≥5	12	Lager	Barely	-
23	N.D.	16.916	China	≥3.1	8	Lager	Barely	Rice
27	130.621	N.D.	China	≥5.2	12.5	Lager	Barely	Highland barley
30	61.393	N.D.	China	≥2.5	8	Lager	Barely	Rice
33	N.D.	31.983	China	≥3.1	8	Lager	Barely	Rice
37	67.030	N.D.	China	≥2.5	8	Lager	Barely	-
41	61.866	N.D.	China	≥3.2	8	Lager	Barely	-
43	N.D.	15.072	China	≥3.1%	7.5	Lager	Barely	Rice, glucose syrup
44	N.D.	11.437	Germany	5.3	12.2	Lager	Barely	-
45	131.470	11.223	China	≥5.2	≥12.5	Lager	Barely	Highland barley
46	N.D.	10.135	China	≥3.1	7.5	Lager	Barely	Rice, glucose syrup
47	N.D.	11.529	China	≥3.2	8	Lager	Barely	Rice
48	N.D.	11.550	China	≥3.1	8	Lager	Barely, wheat	Rice
49	N.D.	11.328	China	≥3.1	8	Lager	Barely	lemon
50	N.D.	10.866	China	≥5	11.2	Lager	Barely	Rice, maize
51	N.D.	9.962	China	≥2.5	8	Lager	Barely	-
52	N.D.	9.654	China	≥3.1	8	Lager	Barely	Rice
53	56.302	8.218	China	≥3.6%	9	Lager	Barely	Rice
54	50.231	9.446	China	≥3.1	8	Lager	Barely	Rice
55	N.D.	8.858	China	≥3.6	10	Lager	Barely	Rice
56	N.D.	8.451	China	0	5	Lager	Barely	Dietary fiber
57	N.D.	98.756	China	≥4.6	12.5	Lager	Barely	-
58	N.D.	9.451	Belgium	9	18.9	Lager	Barely, wheat	-
59	N.D.	8.774	Lithuania	5.5	13.5	Lager	Barely	-
60	N.D.	10.498	Japan	6	12.9	Lager	Barely	Rice, maize
61	N.D.	9.260	Japan	5.5	12	Lager	Barely	Rice, maize

62	N.D.	8.435	Japan	5	11	Lager	Barely	Starch, maize, rice
63	N.D.	9.549	China	≥3.1	8	Lager	Barely	-
84	53.430	N.D.	China	≥4.3	11	Lager	Barely	rice
85	54.678	N.D.	China	≥2.8	11	Lager	Barely	Rice, apple juice
87	78.199	N.D.	China	≥6	16.5	Ale	Barely, wheat	oat
88	61.723	N.D.	China	≥4	11	Ale	Barely	Pineapple juice
90	64.455	N.D.	China	≥5.5	12	Ale	Barely	Flavedo, coriander
91	66.260	N.D.	China	≥4.5	11.5	Ale	Barely	Juice
93	57.509	N.D.	China	≥6.2	14.5	Ale	Barely	-
94	70.567	N.D.	China	≥5.2	14.5	Ale	Barely	-
95	53.534	N.D.	China	≥6	13.5	Ale	Barely, wheat	-
96	59.146	N.D.	China	≥4.8	12	Ale	Barely	-
97	63.688	N.D.	China	≥5	12	Ale	Barely	-
98	71.837	N.D.	China	≥4.8	12.6	Ale	Barely	Jasmine tea
101	56.682	N.D.	China	≥4	11	Ale	Barely	Pineapple juice
103	56.939	N.D.	China	≥5.5	13.4	Ale	Barely, wheat	Mango juice, oat
107	61.905	N.D.	China	≥5.9	15.5	Ale	Barely	-
108	52.220	N.D.	China	≥4.2	10.3	Ale	Barely, wheat	-
112	55.216	N.D.	China	≥5.9	15.5	Ale	Barely	-
113	99.390	N.D.	China	≥8.2	≥18.0	Ale	Barely	Highland barley
114	57.799	N.D.	China	≥6.2	14.5	Ale	Barely	-
115	64.037	N.D.	China	≥6.0	16.5	Ale	Barely, wheat	Oat
116	77.873	N.D.	China	≥6.5	13.5	Ale	Barely, wheat	Orchid
120	51.018	N.D.	China	≥4.2	12	Ale	Barely, wheat	Oat, flavedo, coriander
121	54.641	N.D.	China	≥5	12.6	Ale	Barely	-
122	58.462	N.D.	China	≥4.5	13.5	Ale	Barely	-
128	58.957	N.D.	China	≥5.5	13	Ale	Barely	Honey
132	55.750	N.D.	Belgium	10.5	21.77	Ale	Barely	-
133	51.601	N.D.	Lithuania	5.6	16	Ale	Barely, wheat	Mango juice
139	61.094	N.D.	Lithuania	5	12	Ale	Barely, wheat	-
140	63.800	N.D.	Lithuania	6	14.4	Ale	Barely	-
141	54.652	N.D.	China	≥5	11.8	Ale	Wheat, barely	-
143	54.787	N.D.	Germany	4.9	11.5	Ale	Wheat, barely	-
145	53.536	N.D.	China	5.5	13	Ale	Barely, wheat	Honey
146	51.024	N.D.	China	3.3	10	Ale	Barely, wheat	-
148	50.604	N.D.	Germany	5.5	12.5	Ale	Wheat, barely	-
150	50.484	N.D.	China	≥3.3	9.6	Ale	Barely, wheat	-
152	50.096	N.D.	Germany	5.3	11.5	Ale	Wheat, barely	-
154	59.691	N.D.	China	≥5.9	15.5	Ale	Barely	-
158	52.623	N.D.	Germany	4.7	10.6	Ale	Barely, wheat	-

N.D.=below the LOD

Table S2 The average daily intake of beer in Shanghai and participants for average body weight.

Area	Bw (kg)	Daily intake (L/day)
Total population	58.20	0.078
Adult men	62.70	
Adult women	54.00	
7-10-year-old boys	38.30	
7-10-year-old girls	33.40	

Table S3 Contamination levels of D3G and ZEN in different kinds of beer samples from Shanghai, China (μg/kg).

Beer type	D3G				ZEN				D3G+ZEN
	Incidence (%)	Positive number	Mean (μg/kg)	Range (μg/kg)	Incidence (%)	Positive number	Mean (μg/kg)	Range (μg/kg)	Number
Fermentation style									
Lager	22.4%	19/85	29.28	5-495.24	30.6%	26/85	8.4	5-98.76	5
Ale	49.3%	36/73	32.15	5-99.39	< LOD	< LOD	< LOD	< LOD	0
Alcohol content									
Non-alcohol	< LOD	< LOD	< LOD	< 5	25%	1/4	5.86	5-8.45	0
3-5% vol.	24.14%	21/87	18.86	5-106.89	18.39%	16/87	7.77	5-98.76	3
> 5% vol.	50.75%	34/67	47.38	5-495.24	13.43%	9/67	5.66	5-11.44	2
Beer color									
Pale	39.13%	9/23	26.19	5-70.57	0%	0/23	< LOD	< LOD	0
Amber	31.62%	37/117	28.92	5-495.2	19.66%	23/117	7.35	5-98.76	4
Dark	50%	9/18	47.17	5-131.5	16.67%	3/18	5.79	5-11.22	1
Beer origin									
domestic	36.36%	44/121	27.85	5-131.5	15.70%	19/121	7.12	5-98.76	4
foreign	29.73%	11/37	39.59	5-495.2	18.92%	7/37	5.87	5-11.44	1
Total samples	34.81%	55/158	30.6	5-495.24	16.46%	26/158	6.83	5-98.76	5
